# The QGIS project: Spatial without compromise

**DOI:** 10.1016/j.patter.2025.101265

**Published:** 2025-05-20

**Authors:** Anita Graser, Tim Sutton, Marco Bernasocchi

**Affiliations:** 1 AIT Austrian Institute of Technology, 1210 Vienna, Austria; 2 Kartoza LDA, 7330-151 Lisbon, Portugal; 3 OPENGIS.ch GmbH, 7031 Laax, Switzerland

**Keywords:** open source, geographic information system, spatial data science, data management, spatial data, data analysis, cartography

## Abstract

The QGIS project is a prominent open-source geographic information system (GIS) that has evolved over two decades, contributing significantly to the geospatial community. This paper presents the development, governance, and operational challenges faced by QGIS, providing an in-depth analysis of its growth from a hobby project to a global platform. We examine the project’s organizational structure, release management, and infrastructure, alongside the financial model that sustains its development. The paper also addresses key challenges such as licensing complexities, group decision-making dynamics, and the balancing of innovation with stability in an open-source environment. Additionally, we highlight QGIS’s broad applicability across industries and its continued success in fostering community-driven development.

## Highlights

• QGIS is a leading GIS platform with a global impact
• Its open-source nature makes QGIS a key tool for diverse geospatial applications
• Community-driven development ensures continuous innovation
• QGIS enables open, reproducible research and promotes digital sovereignty

## Teaser

Graser et al. present the evolution, governance, and impact of QGIS, a leading geographic information system (GIS). They highlight its role in democratizing geospatial data management and analysis, enabling open and reproducible research, and fostering digital sovereignty through sustainable open-source development. By examining its governance and development model, this paper underscores how QGIS provides a robust, accessible alternative to proprietary GIS solutions, ensuring that spatial analysis remains transparent, inclusive, and globally impactful.

## Introduction

QGIS is an open-source geographic information system (GIS) that enables users to manage, analyze, and visualize spatial data, providing deeper insights into the world around us. It is widely estimated that approximately 80% of all information contains a geographic component,^1^ whether it relates to specific locations such as addresses, land parcels, or road intersections. While valuable patterns and relationships are often embedded within this geographic data, they can only be effectively uncovered through GIS technology. GIS platforms integrate diverse spatial data formats, including vector data, which stores both the geometric properties of objects and their associated descriptive attributes, as well as raster data such as remote sensing imagery, enabling users to derive valuable insights from satellite and aerial data. For a thorough introduction to GIS, readers are referred to Longley et al.^2^

GIS software is an essential tool for addressing societal and environmental challenges and opportunities, as many of these are inherently spatial in nature. However, the GIS ecosystem is largely dominated by proprietary alternatives, which are often prohibitively expensive and inaccessible to many potential users. The QGIS project seeks to address this gap by providing a high-quality, accessible, and free GIS platform, empowering individuals and organizations to responsibly manage societal and environmental resources.

**Figure 1:**
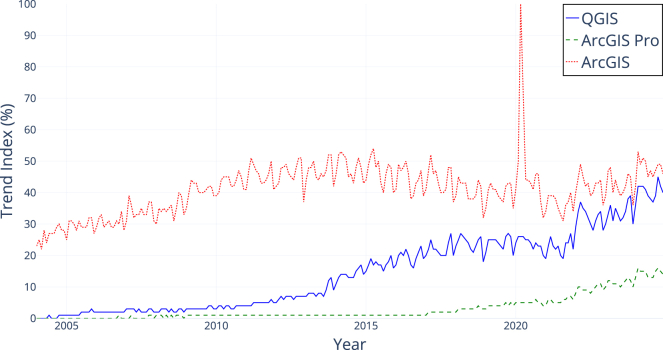
Google search trends comparing QGIS with Esri Source: Google Trends (data provided in the supplemental information).

Development of QGIS began in 2002 as a simple viewer for the open-source spatial database PostGIS (https://postgis.net/). Over time, it has evolved into a comprehensive GIS platform with a global user base. Although quantifying QGIS’s market position is challenging due to a lack of public user statistics for proprietary software and the absence of user registration requirements for QGIS, indicators such as Google Trends (https://trends.google.com/trends/explore?date=all&q=QGIS,ArcGIS%20Pro,ArcGIS) suggest that QGIS is on track to becoming the leading desktop GIS application, if it has not already achieved this distinction (Figure 1).

QGIS is available across a wide range of platforms, including Linux, Windows, and macOS, as well as mobile applications for Android and iOS. Efforts are also under way to develop a browser-based version using web assembly. The software has been translated into over 100 languages, with 20 translations covering more than 75% of the application’s functionality (https://explore.transifex.com/qgis/QGIS/). Regular updates ensure the platform remains robust and cutting-edge, with new versions released every 4 months and long-term releases (LTR) introduced annually. The latter provide a more stable base for enterprise use, receiving only bug fixes and no new features through their lifespan.

The QGIS ecosystem comprises multiple tightly integrated components. QGIS Desktop offers a comprehensive suite of tools for data capture, exploration, advanced cartography, and the automated generation of map series and reports. It supports a wide array of data formats, including vector, raster, point cloud, and mesh datasets. Its powerful processing framework further enables complex spatial analyses and model-building workflows.^3^ QGIS Server, built on the same codebase as QGIS Desktop, functions as a robust and standards-compliant web-mapping platform. It leverages QGIS’s advanced styling capabilities to render high-quality maps dynamically and deliver a range of geospatial services. Supporting key Open Geospatial Consortium (OGC) standards, such as Web Map Service, Web Feature Service, Web Coverage Service, Web Map Tile Service, and OGC application programming interface (API) features, it facilitates seamless integration with a broad spectrum of geospatial clients and workflows.^4^

Beyond its core components, the QGIS ecosystem benefits from a vibrant community effort that maintains auxiliary support services, such as comprehensive documentation, tutorials, and a plug-in repository, as well as infrastructure for automated package building, documentation writing, user support, news, and knowledge sharing.

The project also enables an ecosystem of third-party tools, including mobile data collection solutions (e.g., QField, https://qfield.org/; Mergin Maps, https://merginmaps.com/; and Roam, https://github.com/roam-qgis/Roam) and platforms for publishing QGIS projects online (e.g., LizMap, https://www.lizmap.com/en/; G3W, https://g3wsuite.it/en/g3w-suite-publish-qgis-projects/; and GISQUICK, https://gisquick.org/). Additionally, QGIS.ORG maintains QGIS Web Client 2 (QWC 2).

The governance of QGIS is structured as a transparent and democratic community. A project steering committee (PSC) resolves complex issues, oversees legal and financial management, and provides strategic direction. Key decisions are made through community voting, and all financial reports and outcomes of annual general meetings are published openly on https://qgis.org/community/foundation/finance/. Finally, the legal and financial aspects of the project are managed through the QGIS.ORG Association, a registered entity in Switzerland that is responsible for intellectual property protection, trademarks, and funding management.

This paper provides a detailed overview of these various components and initiatives, illustrating how QGIS continues to advance as an accessible, feature-rich GIS platform for the global community. In the following section, we describe the origins and evolution of the QGIS project, as well as the current ecosystem and QGIS features. Afterward, we provide details on the availability of QGIS and its impact. Finally, we discuss some of the major challenges that the QGIS project faces and how they are being addressed.

## Methods

QGIS’s evolution from a modest open-source initiative to a powerful and versatile GIS platform is rooted in its community-driven development. To understand QGIS’s evolution, this section provides insights into the historical development and current ecosystem of the project, including the technical infrastructure, organizational framework, and financial model that support its continued growth and innovation. Finally, we highlight the key features that make QGIS a powerful and versatile GIS platform.

### QGIS history

#### Origins and early development (2002–2007)

QGIS began in early 2002 as a personal project by Gary Sherman, a software engineer and GIS enthusiast from Alaska. At the time, the geospatial software market was dominated by proprietary tools, which were not only expensive but also limited by restrictive licensing and platform-specific requirements, typically for the Windows operating system. Recognizing the need for a free and open-source alternative, Sherman initiated the development of Quantum GIS (later renamed QGIS).

QGIS was written in C++ and built using the Qt toolkit, enabling cross-platform compatibility. (The Q in the project name is a nod to Qt.) The initial public release, version 0.0.1, debuted in July 2002 with basic GIS functionality, such as map viewing and vector data support. Despite its modest feature set, the project quickly garnered attention within the open-source community, establishing a foundation for future growth.

The project’s progress was driven by an active community of contributors, who began adding features and expanding QGIS’s capabilities. QGIS gained traction among academics and GIS professionals who appreciated its openness, flexibility, and extensibility. During this period, QGIS adopted a community-driven governance model and entered the OSGeo incubation process in March 2007.^5^

The OSGeo incubation process formalized the governance structure, ensuring transparency and community engagement while helping QGIS grow into a mature open-source project. A key milestone during this phase was the establishment of a PSC, which provided a clear framework for decision-making and oversight.

#### Expansion of capabilities and infrastructure (2008–2012)

QGIS graduated from OSGeo incubation in 2008.^6^ In the years that followed, it experienced rapid growth in functionality and infrastructure. A significant milestone was the release of QGIS 1.0 Kore in 2009. This release marked the project’s transition to a stable, feature-rich GIS platform, introducing essential functionalities such as symbology management, raster and vector support, and a robust plug-in architecture.

The plug-in system proved transformative, allowing third-party developers to extend QGIS’s capabilities without modifying the core code base. This approach encouraged innovation and led to the development of a wide array of plug-ins, ranging from geospatial analysis tools to data visualization utilities. By this time, QGIS had gained adoption among public institutions, non-governmental organizations, and professionals, further solidifying its position as a viable alternative to proprietary GIS software.

The first QGIS contributor meetings (previously called “hackfests”) were held in 2009 in Hannover, Germany, and Vienna, Austria. These meetings brought together contributors to collaborate on feature development and strategic planning (including planning for QGIS 2.0).^7^ Additionally, the first official QGIS user group was established in Solothurn, Switzerland, in 2012.^8^

#### The QGIS.ORG association and organizational maturity (2013–2017)

In 2013, the release of QGIS 2.0 Dufour introduced significant architectural and usability improvements, including a refactored code base and a redesigned user interface. This update enhanced the platform’s performance and accessibility, laying the foundation for its modern feature set. It also marked the official transition from the name “Quantum GIS” to “QGIS” to streamline branding and avoid confusion.

To further engage its user base, QGIS began publishing detailed visual changelogs (https://qgis.org/project/visual-changelogs/) with each release, showcasing new features and improvements.

The first international QGIS User Conference was held in 2015 in Nødebo, Denmark.^9^ This event brought together users, educators, and developers to share knowledge and collaborate on advancing the platform.

In 2016, QGIS took a significant step toward sustainability by establishing the QGIS.ORG Association, a Swiss legal entity (Verein im Sinne des Schweizerischen Zivilgesetzbuches [Art. 60–79 ZGB]). This organization was tasked with managing project funds, coordinating efforts, and ensuring long-term stability. This formal structure enabled QGIS to secure sustainable funding, support quality assurance activities, and invest in essential infrastructure work.

#### Transition to QGIS 3 and the path forward (2018–present)

The release of QGIS 3.0 Girona in 2018 marked a major technological leap. This version transitioned the platform to Python 3 and the Qt5 toolkit, ensuring compatibility with modern software standards. The key features of QGIS 3 included enhanced three-dimensional visualization, improved geoprocessing tools, and the introduction of the data source manager for streamlined data handling.

During the COVID-19 pandemic, the QGIS community adapted by launching monthly virtual QGIS Open Day (QOD) events (https://github.com/qgis/QGIS/wiki/QOD-Events). These online gatherings provided a platform for users and contributors to interact, share knowledge, and collaborate. Hosted on the open-source Jitsi platform and live streamed to YouTube (https://www.youtube.com/@qgishome), these events continue to be a valuable resource.

In 2023, QGIS launched its first large-scale crowdfunding campaign,^10^ which brought in 42 new sustaining members, including the first flagship sustaining member. This effort increased annual member contributions to over €200,000,^11^ enabling the project to address long-standing infrastructure needs. Key improvements funded by this initiative included an overhaul of the project website, which launched in July 2024.^12^

Through these ongoing efforts, QGIS continues to evolve as a premier open-source GIS platform, supported by a vibrant global community and a sustainable organizational framework.

### Current ecosystem

The sustainability and development of the QGIS project rely on a well-defined ecosystem that includes formal governance, a consistent release process, reliable infrastructure, and transparent financial management.

#### Organization

QGIS.ORG operates as a legally established association based in Switzerland, with governance designed to promote transparency, collaboration, and democratic decision-making. Its governance structure comprises a Board of three members (chair, vice-chair, and treasurer) who have legal representation, and a PSC of seven members (two of them honorary members) responsible for overseeing project strategy. Both the Board and PSC are elected by the association’s voting members, ensuring a representative and community-driven approach.

Voting members are integral to the governance process, as they elect leadership positions and approve essential project matters. These include the annual chair’s report, financial reports, and the upcoming year’s budget. Voting members also decide on grant proposals and other issues affecting the community. According to the association’s statutes, each country with a QGIS User Group elects a representative, known as a Country User Group Voting Member, who advocates for their country’s interests. To ensure a diverse perspective, one member from the broader community may also be elected for every country voting member. This governance framework underscores QGIS’s commitment to openness, accountability, and fostering a global, inclusive community.

#### Release management

The QGIS project follows a structured and predictable time-based release cycle to deliver regular updates while maintaining high software quality. New releases are scheduled every 4 months, with 3 months dedicated to development and 1 month designated as a “feature freeze” period. During the feature freeze, the focus shifts to testing, bug fixes, translation updates, and release preparations.

QGIS uses an even-odd versioning system: even-numbered versions (e.g., 2.18, 3.2) represent stable releases, while odd-numbered versions (e.g., 2.99, 3.1) are development versions. This system ensures a clear distinction between stable and experimental builds, aiding user adoption and testing efforts.

To cater to educators and organizations that require stability, QGIS also provides an LTR. Every third release is designated as an LTR, with maintenance and updates provided until the subsequent LTR. This approach strikes a balance between innovation and stability, enabling users to plan their workflows effectively while benefiting from consistent software improvements.

#### Infrastructure management

The QGIS project relies on a robust and distributed infrastructure to support its global ecosystem. Core infrastructure includes physical servers hosted in Europe, which provide resources for website and plug-in hosting, system monitoring, feed aggregation, and the management of services used to provide auxiliary services for the QGIS project. These servers also handle automated tasks such as documentation builds, Debian package creation, and automated testing. A dedicated backup server ensures data security and integrity.

Additional infrastructure partners play critical roles in supporting QGIS operations. Kartoza has developed much of the web infrastructure such as the changelog site and training certification platform, while Norbit oversees Windows builds and OPENGIS.ch oversees macOS builds.

The QGIS team is actively transitioning its services to well-documented, monitored systems with enhanced security measures. This ongoing effort underscores the project’s commitment to ensuring a reliable, secure infrastructure to support development, deployment, and user needs.

#### Budget

As a Swiss-based association, QGIS.ORG adheres to stringent financial transparency practices by publishing annual budgets and financial reports (https://qgis.org/community/foundation/finance/). It is important to note that the QGIS.ORG budget reflects only a portion of the global investment in QGIS, as many organizations and businesses contribute directly to its development through consulting and custom implementation services. These external contributions are not included in the QGIS.ORG budget figures, but they significantly enhance the overall ecosystem.

For 2024, QGIS.ORG’s income is projected to reach €395,000, up from €371,000 in €2023 and €214,000 in 2022. According to the latest financial report from 2023, most of the funding (70.6%) comes from sustaining members, followed by donations (18.8%) and contributions through QGIS training certificates (7.6%).

On the expenditure side, the largest allocations in 2023 were for bug fixing (29.7%), the QGIS Grant Program (21.2%), and upstream contributions to libraries like Qt5/Qt6 (8.9%). These expenditures highlight the project’s focus on maintaining software quality, fostering innovation through grants, and contributing to the broader open-source ecosystem.

Efforts to improve infrastructure, such as providing QGIS installers for various operating systems, accounted for 8.9% of expenses. Documentation costs represented 5.9% of the budget in 2023, with plans for increased investment in this area. To address the growing need for high-quality documentation, QGIS.ORG hired a full-time documentation writer in mid-2023. This dedicated role reflects the project’s ongoing commitment to supporting its global user base with up-to-date and comprehensive resources.

### Features

QGIS offers a comprehensive suite of functionalities that make it a versatile tool for geospatial data management, analysis, and visualization, including the following:

(1) Extensive data format support: QGIS can open many vector, mesh, raster, and point cloud formats. It also supports connecting to all popular geospatial database environments, including PostGIS, SQL Server, and Oracle. It also supports modern cloud native formats like vector tiles, GeoParquet, cloud optimized GeoTIFFs, and cloud optimized point clouds.
(2) Powerful data capture tools: QGIS provides an extensive collection of tools for creating and editing data that includes spatial editing of vector, mesh, and point cloud datasets and a powerful visual form designer for creating intuitive data capture interfaces. QGIS also includes a tool for georeferencing digital products, such as scanned paper maps.
(3) Cartography tools: QGIS has comprehensive and powerful cartography tools for map layer styling and labeling. The cartography for vector layers can be controlled with QGIS’s expression language, allowing properties to be dynamically calculated based on logic defined by the cartographer.
(4) Map composition: with the layout composer, QGIS can create high-quality print-ready maps. It can also generate map series/atlas outputs with, for example, one page per county of a national dataset. Advanced users can utilize the report creator, which further allows for the creation of multi-section documents, each with a sub-atlas. Map layouts are also included in the QGIS Server platform, allowing generation of PDF or image outputs via a simple web API.
(5) Processing and analytics tools: QGIS includes an extensive collection of spatial analysis and data processing tools. A powerful visual design tool allows users to create complex, multi-step analysis workflows, which can be saved and shared with other users for replicable analyses.
(6) Programmability: QGIS can be extended in many ways using its Python language bindings. The Python API is well documented, easy to learn, and supports access to nearly every part of QGIS for customization. This has led to the establishment of a rich ecosystem of plug-ins and custom processing algorithms.

While the complete range of features is too extensive to list comprehensively, QGIS has evolved into a general-purpose GIS solution that caters to a wide spectrum of use cases.

#### Supported use cases

Unlike specialized niche tools, QGIS is designed to accommodate diverse workflows across industries, academia, and individual users. The adaptability of QGIS is reflected in its varied applications, which include but are not limited to the following:

(1) Cartography: serving hobbyists and professionals in the creation of high-quality, customizable maps
(2) Forestry, mining, and energy: supporting spatial analysis, resource management, and operational planning
(3) Urban planning and administration: facilitating decision-making and resource allocation in local and regional governance
(4) Environmental protection: enabling monitoring, analysis, and reporting for conservation and sustainability efforts

These examples illustrate the broad utility of QGIS across sectors and disciplines. To showcase its real-world impact, the QGIS project website provides a collection of case studies (https://qgis.org/project/case-studies/) that offer detailed insights into how the software is utilized to address complex geospatial challenges globally.

#### Plug-ins

QGIS core comes with some preinstalled plug-ins: DB Manager, Geometry Checker, GRASS GIS Processing Provider, MetaSearch Catalog Client, OfflineEditing, Processing, and Topology Checker. In addition to these core plug-ins, the QGIS Plug-ins web portal (https://plugins.qgis.org/) hosts over 2,000 plug-ins that provide a variety of additional features to expand QGIS’s core functionality. The most popular plug-ins (with over 1 million downloads each) are focused on

(1) Basemap plug-ins: basemap layers from different web services (e.g., QuickMapServices, OpenLayers Plugin, HCMGIS)
(2) Analytics plug-ins: including supervised classification of remote sensing images (Semi-Automatic Classification Plug-in), vector data analytics (mmqgis), coordinate capture and other convenience functions (Lat Lon Tools), and generation of elevation profiles (Profile tool)
(3) OpenStreetMap plug-ins: providing access to OpenStreetMap (OSM) data (QuickOSM)
(4) Web mapping plug-ins: providing capabilities to export QGIS maps as web maps using Openlayers and Leaflet (qgis2web) and threejs (Qgis2threejs)

Some essential plug-ins of the past have, over time, been integrated into the core, such as, PostGIS Manager and Spatialite Manager (now DB Manager), SEXTANTE (now Processing), Time Manger (now Temporal Controller), Table Manager (now integrated into the attribute table), Vector Tiles Reader (now integrated in the core data source manager), xyToPoint (now available in the Processing toolbox).

### Resource impact and reuse

The QGIS project has a significant global presence, reflected in its widespread user base, active community involvement, and frequent reuse as a foundation for custom applications. It also enables a range of business models that support ongoing development and professional services.

#### User numbers

It is challenging to accurately estimate the number of QGIS users because of the absence of user registration, reliable download metrics, and licensing requirements due to the open-source nature of the QGIS project. For example, some users may download the application multiple times, share installers within organizations, or automate downloads through robots, which do not necessarily result in usage. Moreover, on Linux systems, QGIS is often installed via package managers, further complicating tracking efforts.

To gain insight into the user base, QGIS analytics (https://analytics.qgis.org) collects limited information whenever the software accesses its news feed (https://feed.qgis.org). The aggregated data include the date, QGIS version, operating system (where available), and the user’s country of origin (inferred from the IP [Internet protocol] address). As of October 2024, this platform registered 17,604,358 application starts over a 30-day period. Of these, 49.99% originated from Windows, 2.54% from macOS, 0.26% from Linux, 1.20% from other operating systems, and 46.01% were from older QGIS versions that do not report the operating system.

QGIS users have founded over 30 local user groups (https://qgis.org/community/groups/) in the following countries (at the time of writing): Denmark, Peru, Italy, Scotland, England, Portugal, Wales, Germany, Switzerland, Poland, Brazil, Japan, Norway, South Africa, France, Sweden, Kenya, Australia, United States, Romania, Spain, Colombia, Mexico, the Netherlands, Indonesia, Slovakia, Ecuador, Austria, Ghana, Argentina, North Macedonia, Albania, and Kosovo. Additionally, a global virtual group exists on Facebook. These local groups, alongside individual organizations, play a key role in funding the project. Over 100 sustaining members (https://qgis.org/funding/membership/members/), including private companies, public institutions, and universities, contribute to the QGIS.ORG Association. This funding has a profound impact, enabling access to GIS software for communities in developing regions that could not otherwise afford proprietary solutions.

The first book on QGIS, *Desktop GIS: Mapping the Planet with Open Source Tools*, was published by QGIS founder Gary Sherman in 2008.^13^ Since then, numerous books have been published (https://qgis.org/resources/books/) in English,^14^^,^^15^ Chinese,^16^ Dutch,^17^ French,^18^ Greek,^19^ Italian,^20^ Japanese,^21^ Polish,^22^ Turkish,^23^ and Ukrainian.^24^

For scientific contributions, QGIS offers guidelines for proper citation (https://www.qgis.org/resources/support/faq/). Community events, such as contributor meetings and conferences, play a central role in the QGIS project. Since the first international meeting in Karlsruhe, Germany in 2008, 27 contributor meetings have been organized (https://github.com/qgis/QGIS/wiki#qgis-hackfests). To broaden participation, monthly virtual events, known as QOD (https://github.com/qgis/QGIS/wiki/QOD-Events), began in 2020. These events are recorded and shared on the QGIS YouTube channel (https://www.youtube.com/@qgishome), providing global access to presentations and discussions.

#### Reuse and business models

The QGIS project supports a diverse range of business models and commercial activities, demonstrating its adaptability and economic significance. While precise data on the total economic impact of QGIS are unavailable, it is reasonable to estimate that the project supports a multi-million-dollar ecosystem annually (based on the number of core contributors and commercial service providers on https://www.qgis.org/resources/support/commercial-support/). The key areas of economic activity include the following.

(1) Training: organizations provide courses, tutorials, and other educational materials to build capacity in GIS and QGIS usage.
(2) Consulting: professional services leverage QGIS for tasks such as spatial analysis, map production, and client-specific solutions.
(3) Third-party software: many businesses develop plug-ins or standalone applications based on QGIS. Examples include QField and Mergin Maps for mobile data collection and KADAS Albireo, a user-friendly mapping application focused on drawing, measuring, and terrain analysis (https://github.com/kadas-albireo/kadas-albireo2). These solutions extend QGIS functionality and deliver tailored value to users.
(4) Web hosting: companies offer hosting services and backend solutions for QGIS Server and web-based viewers, facilitating online geospatial applications.
(5) Core development: one of QGIS’s competitive advantages is its ability to engage core developers for custom feature development. After appropriate community consultation, new features can be swiftly incorporated into the platform. Currently, 18 companies employ core contributors, and many others provide related services (https://www.qgis.org/resources/support/commercial-support/).
(6) Stabilization efforts: core developer companies play a critical role in ensuring that QGIS remains reliable and professional grade; thanks to QGIS.org dedicated budget, these activities can be remunerated.
(7) Support and maintenance contracts: core developers provide ongoing support to ensure the stability, security, and performance of QGIS installations, including troubleshooting, updates, and system monitoring and other tasks discussed with clients. This offers reliable, expert-backed support to minimize downtime and operational risks.
(8) Resellers: while the majority of contributors and businesses operate ethically, some actors engage in rebadging QGIS and selling it to uninformed users without providing access to the source code of the original product or for their modifications, or making them aware of the fact that the software is freely available at https://qgis.org.

The success of these diverse business models highlights the strength of QGIS as an adaptable, community-driven platform that fosters innovation while maintaining its commitment to open access and transparency.

## Discussion

The development and sustainability of a large-scale open-source project like QGIS come with various challenges. These challenges span multiple domains, including governance, legal, economic, and technical aspects, each influencing the project’s growth and long-term viability. Governance challenges (challenges 1 and 3) arise from the need to balance community-driven decision-making with strategic direction, while legal issues (challenge 2) primarily stem from licensing complexities and intellectual property considerations. Economic constraints (challenge 4), such as securing stable funding and maintaining infrastructure, directly impact project sustainability. Additionally, technical and organizational challenges, including software maintenance, feature integration, and accessibility for large institutions (challenge 5), require continuous innovation and collaboration. In the following sections, we examine these key challenges in detail and discuss the strategies employed by the QGIS community to address them.

### Challenge 1: Grassroots development model

Building an open-source GIS application that can compete with and, in many cases, surpass proprietary counterparts is no easy task. Open-source development generally follows one of two approaches: “top down,” where a single entity drives funding, vision, and execution, sharing the source code as a benevolent act; or “bottom up,” where funding and direction emerge from individual contributions, with no centralized orchestration of planning or execution.

QGIS follows the latter model, starting with its founder, Gary Sherman, who shared the source code and management early on. This “bazaar” approach, as described by Raymond,^25^ typifies many open-source projects. However, it presents several challenges:

(1) Potential lack of cohesion in user experience and feature selection
(2) Cumbersome decision-making processes that may delay or even prevent resolutions
(3) Risk for contributors funding features that may ultimately be rejected
(4) Independent developers working for disparate clients may not coordinate their efforts to create cohesive feature sets.

Solution: QGIS mitigates these risks through structured processes like the QGIS Enhancement Proposal (QEP) (https://github.com/qgis/QGIS-Enhancement-Proposals), which allows major features to be reviewed before significant resources are committed. A portion of QGIS’s annual budget is allocated to development that promotes cohesion, with funding distributed via QGIS grant proposals.^26^ The PSC also identifies critical initiatives, fundraises when necessary, and provides top-down interventions to guide development when needed.

### Challenge 2: Licensing

QGIS is distributed under the GNU General Public Licenses (GPL) version 2 or later,^27^ a “copyleft” license requiring derivative works to remain under the same license. While beneficial to the community and ecosystem, this licensing model presents challenges:

(1) Education: educating users and other project stakeholders on the nuances of the GPL license
(2) Compatibility: navigating requirements for plug-ins and integration with proprietary services
(3) Licensing compliance: managing infringements and disputes related to licensing
(4) Evolving technological context: adapting to the static nature of the license over time, especially as technological trends shift (e.g., as happened with the “SaaS loophole” in the move toward cloud-based solutions^28^)

For example, in 2004, contributors agreed to an exception allowing linking with any Qt library version.^29^ Today, such changes would be nearly impossible due to the dispersed, ephemeral nature of contributors.

Solution: While the license is static, QGIS focuses on proactive communication, education, and resolving issues when they arise. The community, although large, is vigilant, with knowledgeable users raising concerns to the PSC, which intervenes as necessary.

### Challenge 3: Group decision-making

QGIS operates as a transparent, community-driven project, unlike proprietary software, where decisions are typically made in secret. This democratic model has its own challenges:

(1) Diverse viewpoints: diverse viewpoints and agendas among contributors, businesses, and users
(2) Cultural differences: cultural differences across the global community that may influence participation
(3) Vocal dissent: a risk of discussions being derailed by vocal dissenters, even if their views are not mainstream
(4) Awareness of governance: difficulty ensuring awareness among users of governance and internal decision-making processes

Solution: QGIS employs a “directed democracy” governance model, combining community participation with oversight by the PSC and delegated decision-makers. Examples include policy decisions on the developer discussion mailing list^30^), the QEP process (https://github.com/qgis/QGIS-Enhancement-Proposals), annual general meetings, and the role of maintainers in reviewing contributions. This hybrid model balances inclusivity with pragmatism, enabling effective governance while remaining aligned with strategic goals. Additionally, QGIS actively seeks to understand its user base through intentional communication strategies, despite users not being required to register or participate in traditional sales channels.

### Challenge 4: Budget limitations

For a project of its scope, the QGIS.ORG organization operates on an extremely limited budget. Consequently, for the longest time, QGIS.ORG did not have any paid full- or part-time employees. Instead, all contribution were provided by professionals working for third parties (or on short-term QGIS.ORG-funded contracts) as well as enthusiasts donating their work. This financial restriction has led to several key challenges:

(1) Infrastructure maintenance gaps: essential tasks, such as maintaining bug trackers, build systems, and web services, are often de-prioritized due to a lack of dedicated resources.
(2) Documentation delays: consistently updating and maintaining user and developer documentation remains a challenge due to its time-intensive nature and the absence of permanent staff.
(3) Volunteer fatigue: overreliance on volunteers or contributors who juggle QGIS tasks alongside their professional commitments risks burnout and loss of expertise over time.
(4) Conflicting priorities: volunteers and third-party contributors often prioritize their own or their clients’ goals, leading to misaligned efforts and gaps in critical areas.
(5) Limited capacity for proactive development: the inability to fund long-term or proactive projects resulted in a reactive approach to issues.

**Solution:** The majority of the project’s financial sources originate from a sustaining membership program. (A more detailed breakdown of the funding sources is provided in the budget subsection.) The sustaining membership program is set up in a way that encourages long-term reliable funding to ensure the sustainability of the QGIS project. Members pay an annual membership fee, which contributes to the QGIS.ORG budget. A key priority of QGIS.ORG since the beginning has been to ensure high software quality. Therefore, a significant share of the available funds has been used for bug fixes and usability and performance improvements. To this end, there is a regular paid bug fixing round before each release (three times per year). Through the growing number of larger sustaining members, QGIS.ORG was able to hire its first full-time documentation writer and full-stack developer responsible for maintaining the project’s web infrastructure in 2023.

### Challenge 5: Access to big organizations

One of the key challenges facing QGIS and the broader open-source ecosystem is access to big organizations. Unlike proprietary GIS vendors, QGIS lacks a dedicated marketing and sales department to advocate for its adoption at the executive level within large enterprises and governmental agencies. This absence creates a significant barrier to entry, as decision-makers are often unaware of the capabilities and benefits of QGIS.

Proprietary GIS companies invest heavily in marketing, direct sales, and strategic partnerships to influence stakeholders at the top of the organizational pyramid. QGIS, as a community-driven project, cannot compete on this front due to its decentralized structure and reliance on volunteers and core developer companies, which operate without a unified marketing strategy and encounters the following key challenges in reaching large organizations:

(1) Awareness gap: decision-makers in big organizations may not be aware of QGIS or its capabilities. Proprietary vendors dominate the market narrative, often portraying open-source solutions as less reliable or lacking enterprise-grade support.
(2) Perceived risk: organizations accustomed to proprietary software may perceive open-source solutions as risky, fearing limited support, compatibility issues, or inadequate documentation. Without a dedicated sales team to dispel these myths, QGIS struggles to overcome such objections.
(3) Procurement processes: large organizations typically have formal procurement processes favoring established vendors with proven enterprise track records. QGIS, without a centralized marketing or legal entity to represent it, often falls outside these procurement frameworks.
(4) Enterprise features perception: proprietary companies frequently highlight enterprise-specific features and integrations, while QGIS relies on its community and third-party developers to showcase similar capabilities. This decentralized promotion model lacks the coherence needed to appeal to high-level decision-makers.

Solution: Accessing big organizations requires a strategic shift in how QGIS positions itself. While proprietary vendors have marketing and sales teams to drive adoption, QGIS must rely on its global community, core developer companies, and advocacy efforts to bridge the gap. By empowering these stakeholders and investing in targeted outreach initiatives, QGIS can break through the decision-making hierarchies of large organizations and enhance its position as a competitive GIS solution. A significant step in addressing these challenges was taken in 2024, when a major effort was made to update the official QGIS website. This update aimed to better communicate the platform’s strengths, provide clearer messaging around enterprise-grade capabilities, and address common concerns raised by large organizations. The revamped website serves as a vital tool to help elevate QGIS in the eyes of decision-makers, offering an accessible gateway to its features, success stories, and professional support options.

## Resource availability

### Lead contact

Further information and requests for resources should be directed to and will be fulfilled by the lead contact, Anita Graser (anitagraser@gmx.at).

### Materials availability

This study did not generate new materials.

### Data and code availability

QGIS source code is available at GitHub under the QGIS organization (https://github.com/qgis). The main QGIS repository (https://github.com/qgis/QGIS) is available under the GPL version 2.0 or greater license and has been archived on Zenodo.^31^

## Author contributions

A.G. is the lead author, conceptualized the paper, developed the outline, and distributed responsibilities among the authors. She wrote the introduction and contributed to several other sections of the manuscript. T.S. contributed significantly to the discussion and methods sections. M.B. provided input to the business cases and discussion sections. All authors participated in a thorough review of the entire manuscript to ensure its quality, coherence, and completeness.

## Declaration of interests

All authors are members of the QGIS PSC. A.G. is vice chair of QGIS.ORG and a researcher at the Austrian Institute of Technology in Vienna, Austria. T.S. is an honorary member of the QGIS PSC and co-founded Kartoza, an open-source GIS service provider in South Africa and Portugal. M.B. is chair of QGIS.ORG and CEO and co-founder of OPENGIS.ch, an open-source GIS company in Switzerland.

## Declaration of generative AI and AI-assisted technologies in the writing process

During the preparation of this work, the authors used ChatGPT to improve the writing style of this article. After using this tool, the authors reviewed and edited the content as needed and take full responsibility for the content of the publication.
